# Acute compartment syndrome and fasciotomy after a viper bite in Italy: a case report

**DOI:** 10.1186/s13052-024-01638-5

**Published:** 2024-04-16

**Authors:** Marco Sassoè-Pognetto, Riccardo Cavalcante, Matteo Paonessa

**Affiliations:** 1 Department of Neuroscience “Rita Levi Montalcini”, University of Torino, C.so Massimo d’Azeglio, 52, 10126 Torino, Italy; 2 Centro Emys Piemonte, ELEADE Società Cooperativa a.r.l, Chiaverano, TO Italy; 3 Pediatric Orthopedic Surgery Department, “Regina Margherita” Children’s Hospital, Torino, Italy

**Keywords:** Vipera, Snakebite, Compartment syndrome, Fasciotomy, Case report

## Abstract

**Background:**

Bites caused by European vipers are rare medical emergencies but can occasionally cause life-threatening complications. Viper venom causes local symptoms, which can be accompanied by systemic manifestations in severe cases. The local effects of snakebites include edema and, more rarely, necrosis and compartment syndrome. The consequences of envenomation are often more pronounced in children due to their smaller body size.

**Case presentation:**

We present the case of a 6-year-old girl who experienced multiple viper bites in the lower limb in northwest Italy. The girl received supportive care but progressed to develop compartment syndrome that required emergency fasciotomy. The patient’s condition improved promptly after surgical decompression and administration of antivenom, but full recovery required prolonged hospitalization and rehabilitation.

**Conclusions:**

This case highlights the importance of obtaining a timely assessment of the severity of viper envenomation without delaying the administration of antivenom in most serious cases. The presence of multiple bite marks on the patient is one factor that may help to predict the clinical severity of snakebites and anticipate symptom progression.

## Background

Vipers are the only medically significant snakes in Europe. Envenomation by European vipers is rare [4] but can result in symptoms of varying severity, which may require treatment with antivenom and prolonged hospitalization [6, 8, 12, 23, 27]. The clinical manifestations of envenomation vary depending on the anatomical site of the bite, the amount and characteristics of the venom, and the general condition of the victim. Children are usually considered to be at greater risk due to their smaller body size [5, 16, 20, 24], although some studies have not identified significant differences in the severity of envenomation between adults and children [12, 22].

The venom of vipers produces cytotoxic and hemorrhagic effects that result in a variety of local cutaneous symptoms, such as edema, ecchymosis and, more rarely, necrosis [6, 23]. Compartment syndrome after bites from European vipers is extremely rare but has been reported in both children and adults bitten on their extremities [2, 3, 10, 11, 28, 30, 32]. Without prompt surgical treatment, compartment syndrome causes ischemic damage to nerves and muscle, resulting in irreversible tissue loss [19]. Here, we report a case in which a girl experienced a viper bite, which rapidly progressed to severe compartment syndrome that required fasciotomy of the leg and foot dorsum. The report is based on data obtained from medical records as well as a direct interview with the young patient and her parents.

## Case presentation

**Fig. 1:**
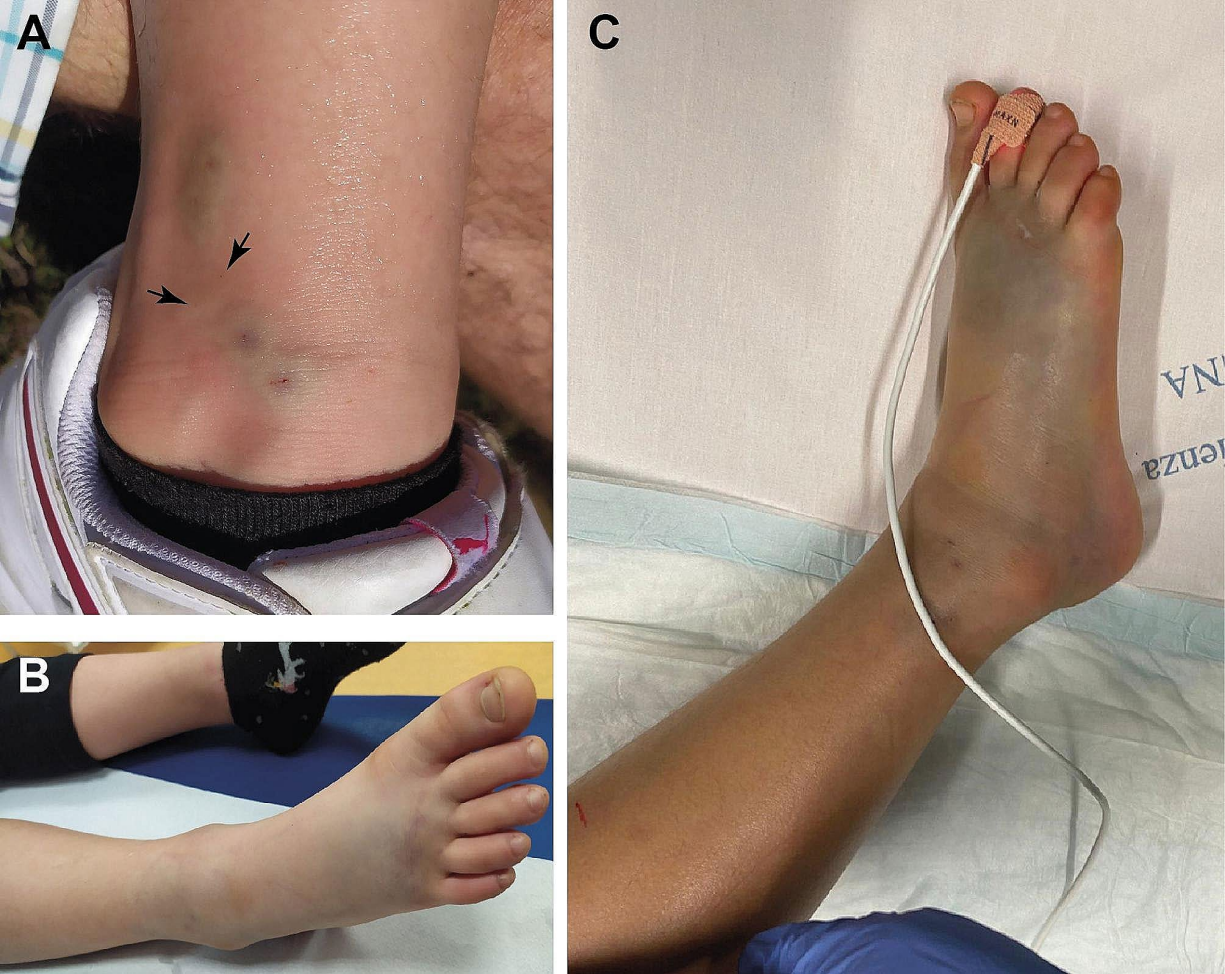
(**A**) Photograph taken shortly after the bite. Two pairs of fang marks are clearly visible and are surrounded by bulges and bruising. Arrows indicate two additional fang marks. Simple measurement with a caliper revealed that the distance between fang marks was 12 mm in the more distal bite, whereas the two other bites had an interfang distance of approximately 8 mm. (**B**) Photograph taken one and a half hours after the bite showing a slightly edematous limb. (**C**) Photograph taken in the operating room approximately nine hours after the bite showing edema and discoloration in the affected limb

A 6-year-old girl with unremarkable medical history was bitten on her right leg on May 24, 2020, at 3:30 p.m. This happened near Bielmonte (Piedmont, Italy), a mountain village located at an elevation of approximately 1500 m. The girl was hiking on a trail lined with small bushes when she felt strong pain on her right ankle. Her parents noticed punctate marks in the proximity of the lateral malleolus and took a photograph (Fig. 1A). Neither the girl nor the parents noticed the presence of snakes or any other venomous animal. However, inspection of the photograph leaves little doubt that the girl was bitten by a viper, as revealed by the presence of two pairs of fang marks with evident edema and bruising, as well as two less evident fang punctures (Fig. 1A). Notably, the distance between the fang tip marks was considerably greater for one bite (12 mm) than for the other (8 mm; Fig. 1A). Two species of viper inhabit the locality where the incident occurred: the common viper (*Vipera aspis*) and the recently described *Vipera walser* [13], an adder whose taxonomic status is still uncertain [9, 29]. Although the species responsible for the envenomation remains undetermined, the habitat in the area (alpine prairie with Rhododendron scrubs) is compatible with the presence of *Vipera walser* (personal observations).

The victim was carried by her father to the car and then transported to the emergency doctor service in Trivero, where the physician assumed the occurrence of a snakebite and recommended swift hospitalization. The girl and her parents reached the closest hospital in Biella at 5:02 p.m., which was an hour and a half after the accident. The drive to the hospital was uneventful, except for a tendency toward drowsiness of the victim.

Upon admission to the emergency department, the girl was alert, with no fever and no signs of nausea or vomiting. The pulse rate was 100 bpm, and the blood pressure was 99/60 mm Hg. The right foot and ankle were slightly edematous and aching (Fig. 1B), and the right thigh and groin were painful to palpation. No neurological defects were observed. The patient was admitted to the short-stay observation unit and treated with paracetamol (250 mg), rehydration therapy and a topical application of gentamicin and betamethasone. At 5:24 p.m., there was a consultation with the Pavia Poison Center, which advised blood tests with D-dimer levels to monitor coagulation. Blood parameters became available at 7:16 p.m., revealing neutrophilic leukocytosis and elevated D-dimer (Table 1).

**Table 1:** Selected hematological parameters

		Reference range	Values
RBC	4.1–5.3 × 10^6^/mm^3^		4.96
HGB	11.6–14.4 g/dl		13.4
WBC	3.5–11.9 × 10^3^/mm^3^		**14.14**
Neutrophils	31.0–65.0%		**73.2**
Lymphocytes	24.0–56.0%		**19.7**
Monocytes	2.0–8.0%		6.5
Eosinophils	0.2–7.1%		0.5
Basophils	0.1–1.3%		0.1
Platelets	180–380 × 10^3^/mm^3^		303
INR	0.80–1.20		0.97
aPTT ratio	0.80–1.20		0.95
Fibrinogen	150–400 mg/dl		212
Antithrombin III	70–120%		86
D-dimer	< 500 µg/L FEU		**784**
C-reactive protein	0-0.50 mg/dl		0.04
CK	26–140 IU/l		**147**
Creatinine	0.20–0.70 mg/dl		0.55

Parameters were evaluated at the emergency department approximately three hours after the bite. Abnormal parameters are highlighted in bold.

 As a result, the Pavia Poison Center recommended treatment with antivenom, which, however, was not administered. One hour later, the clinical condition worsened. The patient became drowsy and had bilateral palpebral edema and an extension of the edema in the foot and leg, with impaired foot dorsiflexion and the absence of appreciable arterial pulses. Chlorpheniramine was given intravenously (7.5 mg in 100 ml). An eco-color Doppler test was performed, with no indication for urgent vascular surgery or fasciotomy. Following anesthesia consultation, the girl was endotracheally intubated and subjected to mechanical ventilation. Arrangements were made for transfer to the Regina Margherita Children Hospital in Torino, and the patient was discharged at 10.34 p.m. after testing negative for SARS-CoV-2.

**Fig. 2:**
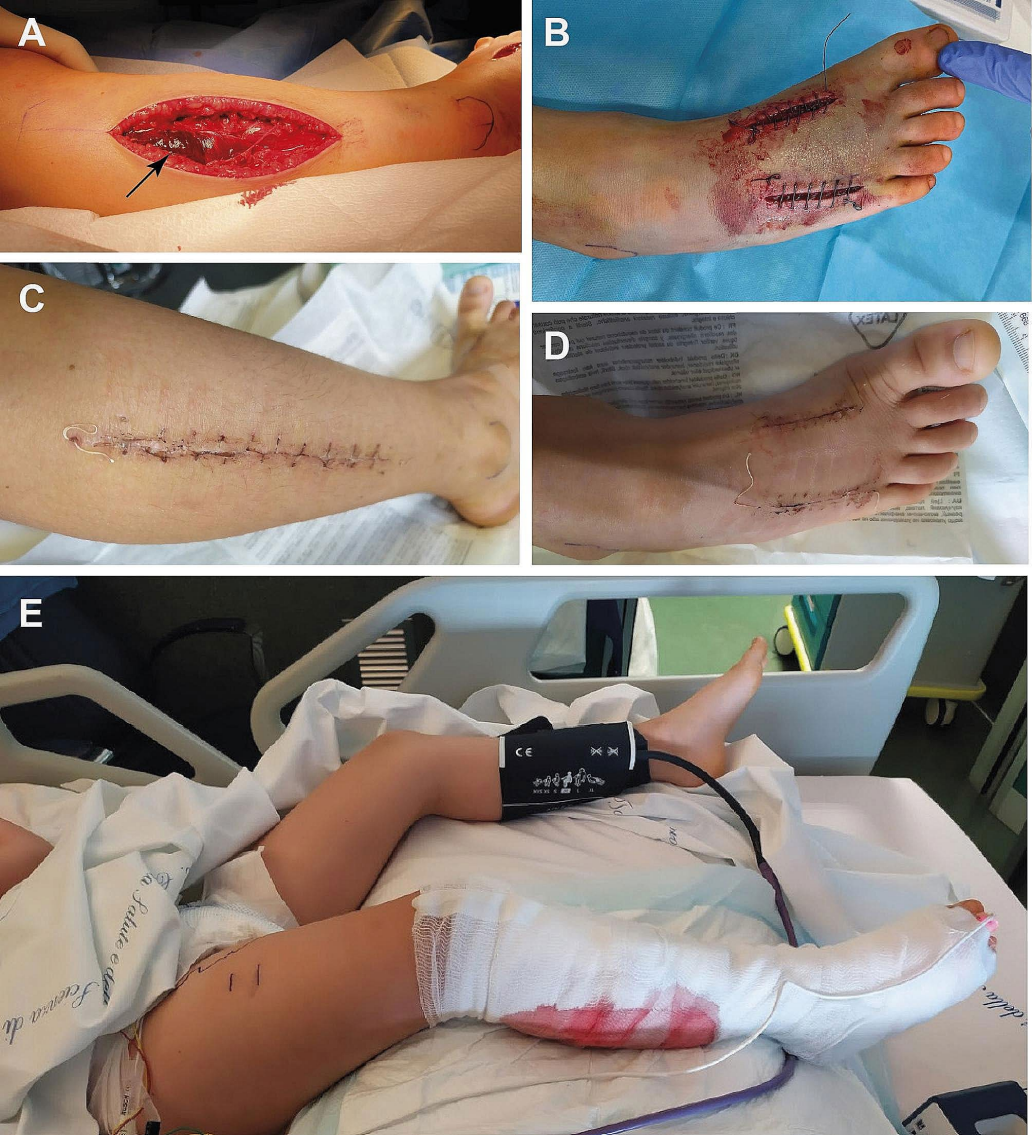
(**A**) Fasciotomy of the leg lateral compartment. Notice the dark color of muscles (arrow), suggestive of initial ischemic damage. (**B**) Fasciotomy of the foot dorsum. (**C**, **D**) Definitive skin closure after 18 days. (**E**) Photograph taken after extubation. Notice the presence of massive edema in the right limb

**Fig. 3:**
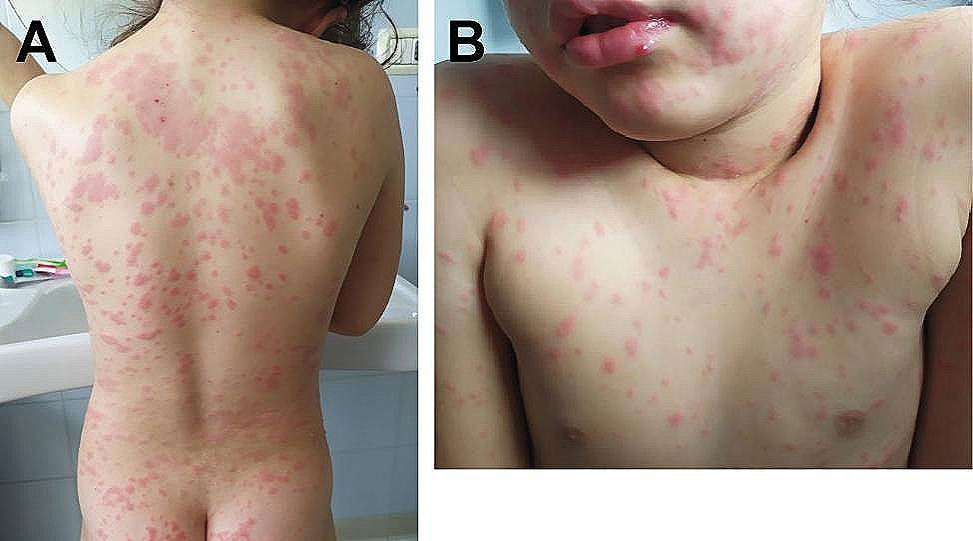
Extensive erythematous papular rash on the back (**A**), thorax and face (**B**) of the young patient

The girl was admitted to the pediatric intensive care unit at 00:05 a.m. on May 25. A venous blood test revealed leukocytosis (total white blood cells: 20.17 10^9^/l, range: 5.00-13.50), hyperglycemia (149 mg/100 ml, range: 70–100), and strongly elevated p-D-dimer (25503 ng/ml FEU, range: <500). Renal and liver functions were largely normal, with slightly elevated urea (48 mg/100 ml, range: 10–43) and creatinine (0.48 mg/100 ml, range: 0.29–0.46). The right foot and leg showed prominent edema and ecchymosis (Fig. 1C). After consultation with the Pavia Poison Center, one ampoule of viper antivenom (Viekvin, Institute of Virology, Vaccines and Sera “Torlak”, Belgrade, Serbia) was administered. This antivenom is made of equine immunoglobulin fragment F(ab’)2 raised against the venom of *Vipera ammodytes* and *Vipera berus*. The patient was also treated with antibiotics (piperacillin and tazobactam 2.5 g × 4; vancomycin 300 mg × 4), antimycotics (fluconazole 200 mg/day), dexamethasone (3 mg × 2), furosemide (15 mg), paracetamol (250 mg × 3), and enoxaparin (200 UI × 2). Moreover, compartment syndrome was diagnosed based on both clinical and instrumental criteria. Specifically, a differential pressure (ΔP = diastolic blood pressure– absolute compartment pressure; McQueen and Court-Brown [26] of < 25 mmHg was recorded in the lateral leg compartment. The patient was taken to the operating room at 1:10 a.m., and fasciotomy was performed with an incision on the dorsal surface of the foot medial to the second metatarsal and lateral to the fourth metatarsal, as well as on the lateral aspect of the leg (Fig. 2). A prominent edematous imbibition was found in the subcutaneous tissue, with ischemic suffering of muscles (Fig. 2A). The postoperative outcome was good, and the girl was extubated the next day and subsequently transferred to the emergency pediatrics department on May 27. During the subsequent course, there was a progressive reduction in edema and pain in the suffering limb. Dressings were changed regularly until definitive closure on June 12 (Fig. 2C, D). On May 30, an eco-Doppler ultrasound exam was performed due to the presence of a painful fibrotic cord on the medial aspect of the right thigh, with no evidence of thrombotic phenomena. Starting on June 13, the girl developed an intensely itchy, erythematous papular rash covering the entire body, compatible with drug-induced toxidermia (Fig. 3). After dermatological consultation, this condition was successfully treated with antihistamines and betamethasone. On June 10, the patient began to perform protected weight-bearing exercises with crutches, and she started walking without the support of crutches on June 18. She was discharged the next day. The patient and her parents reported no significant sequelae when interviewed three years after the incident.

## Discussion and conclusions

The use of fasciotomy for the treatment of snakebites has generated substantial controversy in the clinical literature [14, 17, 19, 25, 31, 33]. One source of ambiguity is that snake envenomation causes hemodynamic instability and severe pain, which mimic the main signs of compartment syndrome. Clinical experience and controlled animal studies have demonstrated that increased compartment pressure can be effectively treated with appropriate administration of antivenom [15, 17]. Accordingly, current guidelines suggest that fasciotomy should be performed only when compartment syndrome persists after adequate antivenom therapy has been administered [7, 21]. Türkmen and Temel [31] proposed an algorithm to guide surgical management and avoid unnecessary fasciotomy in the case of snakebites. On the other hand, a retrospective analysis of 79 young patients in Costa Rica revealed that early fasciotomy reduced secondary infections and significantly shortened hospitalization, suggesting that, in the case of compartment syndrome, one of the most important factors determining patient outcome is the critical timing of surgical intervention [1]. In the present case, the administration of antivenom was delayed, and the patient’s condition rapidly progressed, with intracompartmental pressure increasing to abnormal levels. Therefore, fasciotomy was performed to prevent further complications and potential limb loss.

Compartment syndrome is a rare complication of snakebite. This condition has been reported in 1.4% of 219 cases of viper bite in Switzerland [12] and 1.36% of 147 cases in Greece [11]. Risk factors for developing compartment syndrome after snakebite include leukocytosis and elevated aspartate aminotransferase [19]. In general, high white blood cell (WBC) counts combined with an increased incidence of neutrophils and an increased INR are significantly correlated with the clinical severity of viper envenomation in children [24]. In the present case, the major hematological alterations were neutrophilic leukocytosis and elevated D-dimer levels. However, the patient did not present with systemic symptoms, such as hypotension or gastrointestinal manifestations, which are frequently observed after bites by European vipers [12, 27]. Despite this, five hours after the bite, there was a rapid worsening of the clinical condition, with extension of the edema and increased drowsiness, and the patient was intubated. This highlights the importance of obtaining a timely assessment of the severity of envenomation in the case of a viper bite without delaying immunotherapy, given the potentially rapid diffusion of the venom and progression of the symptoms.

One factor that might have been responsible for the severe symptoms of the present case is the fact that the girl had multiple bites, which might have resulted in the inoculation of an unusually large amount of venom. This situation is quite exceptional and not easily reconciled with the fact that the victim was not aware of the presence of the snake. Possibly, the girl stepped inadvertently on a viper that was concealed under vegetation. Springtime is the mating time for vipers and adders, supporting the possibility that the girl could have received bites from two distinct individuals. This is supported by the difference in the distance between fang marks at bite sites (Fig. 1A), although interfang distance can vary among different bites delivered by the same snake. Notably, the distance between bite marks on the victim can be used to estimate snake size, which is a factor that can help predict the clinical severity of envenomation [18].

Bites from vipers are an uncommon event in Italy and other European countries and in most cases are not life-threatening. However, viper envenomation requires constant monitoring for evolving local and systemic complications and prompt administration of antivenom in severe cases. Fasciotomy is indicated when compartment syndrome develops or persists after hemostatic abnormalities have been corrected by antivenom therapy. Pediatric patients with bites on their extremities and a high WBC count are particularly at risk for developing serious complications, including compartment syndrome. Given the importance of the amount of injected venom in predicting the clinical severity of snakebites, physicians should carefully examine fang punctures at bite sites. The presence of multiple bite marks has significant predictive value for clinical severity. Accordingly, these findings should be reported to poison centers and taken into consideration when planning adequate therapy.

## Data Availability

The datasets analyzed during the current study are available from the corresponding author on reasonable request.

## Abbreviations

aPTT: Activated Partial Thromboplastin Time
CK: Creatine kinase
ΔP: Differential pressure
HGB: Hemoglobin
INR: International normalized ratio
RGB: Red blood cell
SARS-CoV-2: Severe acute respiratory syndrome coronavirus 2
WBC: White blood cell
